# Baicalin Improves Cardiac Outcome and Survival by Suppressing Drp1-Mediated Mitochondrial Fission after Cardiac Arrest-Induced Myocardial Damage

**DOI:** 10.1155/2021/8865762

**Published:** 2021-02-01

**Authors:** Jun Wu, Hui Chen, Jiahong Qin, Nan Chen, Shiqi Lu, Jun Jin, Yi Li

**Affiliations:** ^1^Department of Ultrasonography Medicine, Suzhou Hospital of Traditional Chinese Medicine, 215009 Suzhou, China; ^2^Suzhou Research Institute of Traditional Chinese Medicine, Suzhou Hospital of Traditional Chinese Medicine, 215009 Suzhou, China; ^3^Department of Emergency Medicine, Traditional Chinese Medicine Hospital of Kunshan, 215300 Kunshan, China; ^4^Department of Intensive Care Unit, The First Affiliated Hospital of Kunming Medical University, 650032 Kunming, China; ^5^Department of Intensive Care Unit, The First Affiliated Hospital of Soochow University, 215006 Suzhou, China

## Abstract

Myocardial injury after cardiac arrest (CA) often results in severe myocardial dysfunction and death involving mitochondrial dysfunction. Here, we sought to investigate whether baicalin, a natural flavonoid compound, exerts cardioprotection against CA-induced injury via regulating mitochondrial dysfunction. We subjected the rats to asphyxia CA after a daily baicalin treatment for 4 weeks. After the return of spontaneous circulation, baicalin treatment significantly improved cardiac function performance, elevated survival rate from 35% to 75%, prevented necrosis and apoptosis in the myocardium, which was accompanied by reduced phosphorylation of Drp1 at serine 616, inhibited Drp1 translocation to the mitochondria and mitochondrial fission, and improved mitochondrial function. In H9c2 cells subjected to simulated ischemia/reperfusion, increased phosphorylation of Drp1 at serine 616 and subsequently enhanced mitochondrial Drp1 translocation as well as mitochondrial fission, augmented cardiomyocyte death, increased reactive oxygen species production, released cytochrome c from mitochondria and injured mitochondrial respiration were efficiently improved by baicalin and Drp1 specific inhibitor with Mdivi-1. Furthermore, overexpression of Drp1 augmented excessive mitochondrial fission and abolished baicalin-afforded cardioprotection, indicating that the protective impacts of baicalin are linked to the inhibition of Drp1. Altogether, our findings disclose for the first time that baicalin offers cardioprotection against ischemic myocardial injury after CA by inhibiting Drp1-mediated mitochondrial fission. Baicalin might be a prospective therapy for the treatment of post-CA myocardial injury.

## 1. Introduction

Despite great efforts to improve resuscitation techniques, mortality and morbidity worldwide due to cardiac arrest (CA) remain high [1–3]. Most patients with the return of spontaneous circulation (ROSC) suffer from post-CA myocardial injury. This injury is different from previous myocardial ischemic injury such as myocardial infarction and characterized by persistent myocardial stunning after ROSC, which results in cardiac dysfunction and death after initial resuscitation [4]. Therefore, the promising strategy to reduce myocardial injury after CA is essential and of great value.

Mitochondrial dysfunction serves a pivotal role in myocardial ischemia/reperfusion (I/R) injury pathogenesis after CA [5, 6]. Recent studies have demonstrated that maintaining normal mitochondrial function depends on the dynamic balance of mitochondrial fusion and fission [7, 8]. Mitochondria continuously change their number and shape through fusion and fission, and this process frequently switches to a fission state under stress conditions. Excessive mitochondrial fission has been considered detrimental and is responsible for the impaired energy metabolism, elevated reactive oxygen species (ROS), and released proapoptotic factor. Increasing evidence indicates that mitochondria undergo fission in the heart after I/R injury, and repressing mitochondrial fission prevents cardiac injury and improves myocardial function [9–11]. Mitochondrial fission develops, and its suppression has been documented to attenuate ischemic injury in the heart and brain and improve outcomes after CA [12, 13]. Inhibiting mitochondrial fission might be an attractive therapeutic target for the maintenance of mitochondrial homeostasis and cardiac function after CA.

Baicalin, a natural flavonoid compound isolated from the roots of perennial herb *Scutellaria baicalensis Georgi*, is known as a medical plant used all over the world. Emerging studies have demonstrated that baicalin possesses multiple pharmacological effects constituting anti-inflammatory [14], antiviral [15], anticancerous [16], and antioxidative activities [17]. It has been reported that baicalin can alleviate not only focal but also repeated cerebral ischemic injury [18, 19]. Recent studies have further demonstrated that baicalin improves left ventricular function in a Langendorff model of cardiac ischemia and attenuates cardiomyocyte injury under hypoxia/reoxygenation condition [20, 21]. In addition, baicalin has been initially shown to be effective in preventing mitochondrial damage [22]. However, it is unknown whether baicalin plays a defensive effect against CA-induced myocardial I/R injury and mitochondrial dysfunction via affecting mitochondrial fission.

The function of baicalin in the regulation of mitochondrial fission and modulation of mitochondrial dysfunction-correlated myocardial injury resulting from CA has not yet been studied. Herein, our data showed that baicalin treatment significantly attenuated CA-induced myocardial injury, profoundly facilitated cardiac function recovery and survival, and improved mitochondrial function. Furthermore, inhibition of excessive mitochondrial fission played a pivotal role in mediating baicalin's cardioprotection.

## 2. Materials and Methods

### 2.1. Animals

Male Sprague-Dawley rats weighing 320-380 g were purchased from the Experimental Animal Center of Soochow University (Soochow, China) and maintained fasting for 8-12 hours before beginning the experiment. Research procedures followed the National Institutes of Health Guide for the Care and Use of Laboratory Animals and were approved by the Institutional Animal Care and Use Committee. Furthermore, experiments were reported in compliance with the ARRIVE guidelines and conform to the relevant ethical guidelines for animal research.

### 2.2. CA Model

**Figure 1:**
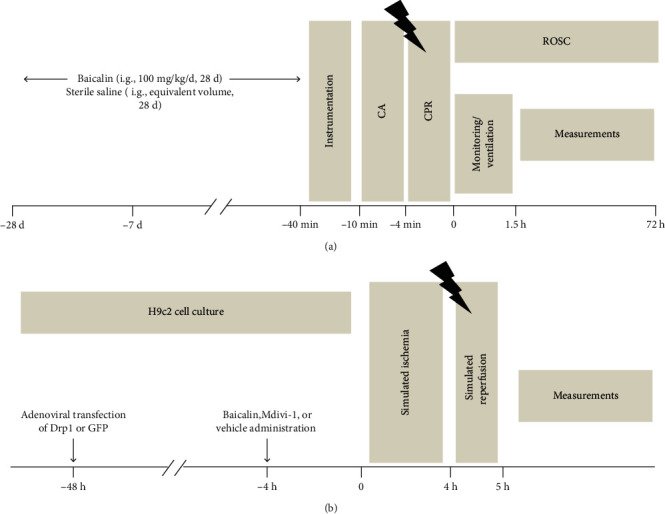
Schematic representation of the experimental protocol. (a) Experimental procedures for the in vivo rat model of cardiac arrest. (b) Experimental procedures for the in vitro cardiac myocytes subjected to ischemia-reperfusion. CA: cardiac arrest; CPR: cardiopulmonary resuscitation; Drp1: dynamin-related protein 1; ROSC: return of spontaneous circulation; GFP: green fluorescent protein.

CA was induced by asphyxia as previously described with a slight modification [23]. Briefly, animals were orotracheally intubated after they were intraperitoneally administered 45 mg/kg pentobarbital sodium. Intravascular catheters were advanced through the left femoral artery and external jugular vein for mean arterial pressure (MAP) monitoring and drug delivery. A 23-gauge polyethylene catheter was advanced from the right carotid artery into the left ventricle for invasive hemodynamic monitoring. After instrumentation, rats were initiated CA by clamping the endotracheal tube. CA was defined as a mean arterial pressure of ≤20 mmHg. At the end of the 6-minute untreated CA, cardiopulmonary resuscitation (CPR) was initiated by mechanical ventilation and chest compressions. ROSC was defined as the presence of an organized supraventricular rhythm with a MAP ≥ 60 mmHg lasting at least 5 minutes. Rats that failed to maintain ROSC within a 1.5-hour observation period were excluded. A schematic illustration of the experimental protocol is shown in Figure 1(a).

### 2.3. Drug Administration and Experimental Groups

After achieved ROSC, animals were divided randomly into two groups: (1) CA group, which underwent CA and received only a vehicle; (2) CA+Bai group, which were subjected to CA and treated with baicalin. In addition, animals underwent the same procedures except those with induced CA were used as the sham group. Baicalin was purchased from Sigma-Aldrich (St. Louis, MO, USA) and dissolved in sterile saline and 100 mg/kg was administered intragastrically once daily for 4 weeks, while rats in the sham and CA groups received an equivalent volume of sterile saline. The last gavage was performed 24 hours prior to induction of CA. As shown in Figure S1, the dose of 100 mg/kg was determined as the effective dose in animals.

### 2.4. Echocardiography

Transthoracic echocardiography was measured using a GE VIVID 7 ultrasound system (GE Healthcare, Milwaukee, WI, USA). Rats were anesthetized with 3% vaporized isoflurane, and 2D left ventricular images were obtained from M-mode echocardiograms. M-mode images were used to measure left ventricular interventricular septum, left ventricular posterior wall thickness, left ventricular dimensions, and left ventricular end-diastolic/systolic volume and to calculate left ventricular ejection fraction (EF) and fractional shortening (FS) [24]. Myocardial performance index (MPI), combining systolic and diastolic time intervals, was calculated as (*A*–*B*)/*B*, where *A* is the mitral valve closure-to-opening interval and *B* is the left ventricular ejection time.

### 2.5. Determination of Serum Creatine Kinase MB and Cardiac Troponin I

Intravenous blood was drawn, and serum creatine kinase MB (CK-MB) and cardiac troponin I (cTnI) were determined using a rat CK-MB ELISA and cTnI ELISA kit (Cusabio, Wuhan, China) according to the manufacturer's instructions, respectively. The assay was performed using a microplate reader (Molecular Devices, Sunnyvale, CA, USA).

### 2.6. Histological Examination

Left ventricular tissues were fixed with 4% paraformaldehyde overnight and embedded in paraffin, and 6 *μ*m thick sections were cut and stained with hematoxylin-eosin (HE) (Beyotime, Shanghai, China) for morphological observation. Terminal deoxynucleotide transferase-mediated dUTP-biotin nick-end labeling (TUNEL) assay (Roche, Mannheim, Germany) was performed using an in situ apoptosis detection kit, as per the manufacturer's instruction. Six fields of vision were randomly selected from each tissue sample, and TUNEL-positive cells were counted under an optical microscope. Apoptosis index was expressed as the number of TUNEL-positive cells/the total number of cells counted × 100%.

### 2.7. Measurement of Caspase-3 Activity

Caspase-3 activity was determined by measuring the generation of the fluorogenic cleavage product methylcoumarylamide using a caspase-3 activity assay kit (Beyotime, Shanghai, China) according to the manufacturer's instructions. Briefly, heart tissues were homogenized in an ice-cold buffer and then centrifuged at 1000g for 5 min. After centrifugation, the supernatant was transferred to a new tube for caspase-3 activity testing. Fluorescence from a 100 *μ*l sample was assayed in fluorescent spectrophotometry together with 100 *μ*l of detection buffer and was then normalized by protein concentration.

### 2.8. Detection of Mitochondrial Morphology

Tissue samples from the ventricular anterior wall were fixed in 2.5% glutaraldehyde (pH = 7.4) overnight at 4°C and 1% osmium tetroxide and dehydrated, embedded in epon, and sectioned. Sections were stained with uranyl acetate and lead citrate and viewed under a transmission electron microscope (FEI, Hillsboro, OR, USA) at a final magnification of ×18,500. Mitochondrial aspect ratio, size, and number were analyzed with Image-Pro Plus software (Media Cybernetics, Rockville, MD, USA). Mitochondria were evaluated in a blind manner about group assignment. Ten randomly selected fields of micrograph were assessed.

Mitochondrial morphology was evaluated in H9c2 cells that were incubated with 100 nM MitoTracker Green probe (Thermo Fisher Scientific, Waltham, MA, USA) for 30 min at 37°C. Images were acquired using a confocal laser scanning microscope (FV 1000, Olympus, Tokyo, Japan). The percentage of cells with fragmented mitochondria (small and round) was determined.

### 2.9. Mitochondrial Isolation

Mitochondria were isolated from the hearts or H9c2 cells as described elsewhere [25]. Briefly, mitochondria were isolated by differential centrifugation and finally resuspended in mitochondrial isolation buffer using a Mitochondria Isolation Kit for Tissue and Cultured Cells (Abcam, Cambridge, UK) according to the manufacturer's instructions [26]. Protein concentration was determined by a BCA protein assay kit (Beyotime, Wuhan, China). Fresh mitochondria were used for detecting mitochondrial respiration and protein expression.

### 2.10. Measurement of Mitochondrial Respiration

The mitochondrial respiratory function was measured by using an oxygen electrode (Oxygraph, Hansatech Instruments, Norfolk, UK). Mitochondria were added to the respiration buffer (GENMED, Boston, MA, USA), and respiration was initiated by pyruvate and malate addition. After 2 minutes, adenosine diphosphate (ADP; 0.5 mmol/l) was added to induce state 3 respiratory rate. Under the conditions of ATP depletion, state 4 rate was determined. Respiratory control ratio (RCR) was calculated by dividing state 3 by state 4 oxygen consumption rates, which demonstrates the tightness of the coupling between mitochondrial respiration and phosphorylation.

### 2.11. Cell Culture and Transfection

H9c2 cardiac cells were purchased from Shanghai Institutes for Biological Sciences (Shanghai, China). Cells were maintained in Dulbecco's modified Eagle's medium (DMEM) containing low glucose with 10% fetal bovine serum supplement and 1% penicillin/streptomycin solution at 37°C in a humidified 5% CO_2_ incubator. To simulate I/R injury in vitro, H9c2 cells were subjected to hypoxia/reoxygenation. Cells that reached 80-90% confluence were switched to DMEM without serum and glucose and incubated in a trigas incubator with 95%N_2_ and 5% CO_2_ for 4 h to induce hypoxia. Then, the cells were cultured for 1 h with the original medium under the normoxic condition at 37°C to reoxygenate. The cells treated with vehicle or baicalin were incubated for 4 hours prior to hypoxia/reoxygenation.

For adenovirus overexpression, H9c2 cells were incubated with recombinant adenoviruses expressing rat dynamin-related protein 1 (Drp1) (GenScript, Nanjing, China). Adenoviruses with a human green fluorescent protein (GFP) were used as negative control. All experiments were performed after 48 h of adenoviral infection (Figure 1(b)).

### 2.12. Determination of Cell Viability and Apoptosis

Cell viability was determined with the Cell Counting Kit-8 (MCE, Monmouth Junction, NJ, USA) according to the manufacturer's instruction. Briefly, H9c2 cells were seeded in a 96-well plate at 5 × 10^3^ cells/well and incubated with various cultural conditions as mentioned above. Following 4 h of incubation at 37°C, CCK-8 solution was added and the absorbance was measured at 450 nm wavelength by a microplate spectrophotometer. Cell apoptosis was assessed using the Annexin V-FITC Apoptosis Detection kit (eBioscience, San Diego, CA, USA) by flow cytometry as previously described [27]. H9c2 cells were trypsinized by 0.25% trypsin and washed in PBS, and cells were centrifuged and resuspended, later stained with Annexin V according to the manufacturer's instructions.

### 2.13. Intracellular ROS Assay

The production of ROS in the H9c2 cells was detected using 2′,7′-dichlorofluorescein diacetate (DCFH-DA; Beyotime, Shanghai, China) probe. After various treatments, H9c2 cells were incubated with DCFH-DA (10 *μ*M) and stained in the dark at 37°C for 30 min. The fluorescence image was then captured by a fluorescence microscope, and the intensity of fluorescence was calculated by ImageJ software.

### 2.14. Western Blotting Analysis

Protein was extracted from fresh tissues or H9c2 cells and placed in lysis buffer to measure total protein expression as previously described [28]. For the analysis of mitochondrial protein expression, mitochondrial fractions were isolated using the mitochondria isolation kit as described above. Samples (approximately 40 *μ*g) were separated on 10% SDS-polyacrylamide gel, and then, the proteins were transferred to polyvinylidene difluoride membranes, which were blocked with 5% nonfat milk and 1% bovine serum albumin and 0.1% Tween-20 in Tris buffer saline for 1 h. The membranes were incubated with primary antibodies against dynamin-related protein 1 (Drp1) (diluted 1 : 1000; Abcam), p-Drp1 (Ser 616) (diluted 1 : 1000; Abcam), mitochondrial dynamics protein of 49 kDa (Mid49) (diluted 1 : 1000; Thermo Fisher Scientific), mitochondrial fission protein 1 (Fis1) (diluted 1 : 1000; Thermo Fisher Scientific), cytochrome c (diluted 1 : 1000; Cell Signaling Technology), voltage-dependent anion channel 1 (VDAC1) (diluted 1 : 1000; Cell Signaling Technology), and *β*-actin (diluted 1 : 1000; Cell Signaling Technology). Next, the membranes were subjected to three 15 min washes with 0.1% Tween-20 in Tris buffer saline and incubated with horseradish peroxidase-conjugated secondary antibody (1 : 2000 dilution) for 45 min at room temperature. The protein bands were detected using an enhanced chemical luminescence system (Cell Signaling Technology, Danvers, MA, USA). VDAC1 was used as loading control for mitochondria. *β*-Actin was used as loading control for whole cell lysis or cytoplasm.

### 2.15. Statistical Analysis

Data were presented as mean ± SEM, and statistical analyses were performed using SPSS 23.0 (SPSS, Chicago, IL, USA) and GraphPad Prism 5.0 (GraphPad, La Jolla, CA, USA). All measured data were analyzed with one-way or two-way analysis of variance followed by Tukey's post hoc tests between multiple experiment groups. The difference in the survival rate between groups was compared by Kaplan-Meier survival analysis with the log-rank test. All *p* < 0.05 was considered statistically significant.

## 3. Results

### 3.1. Baicalin Improved Cardiac Performance and Survival in Post-CA Rats

**Figure 2:**
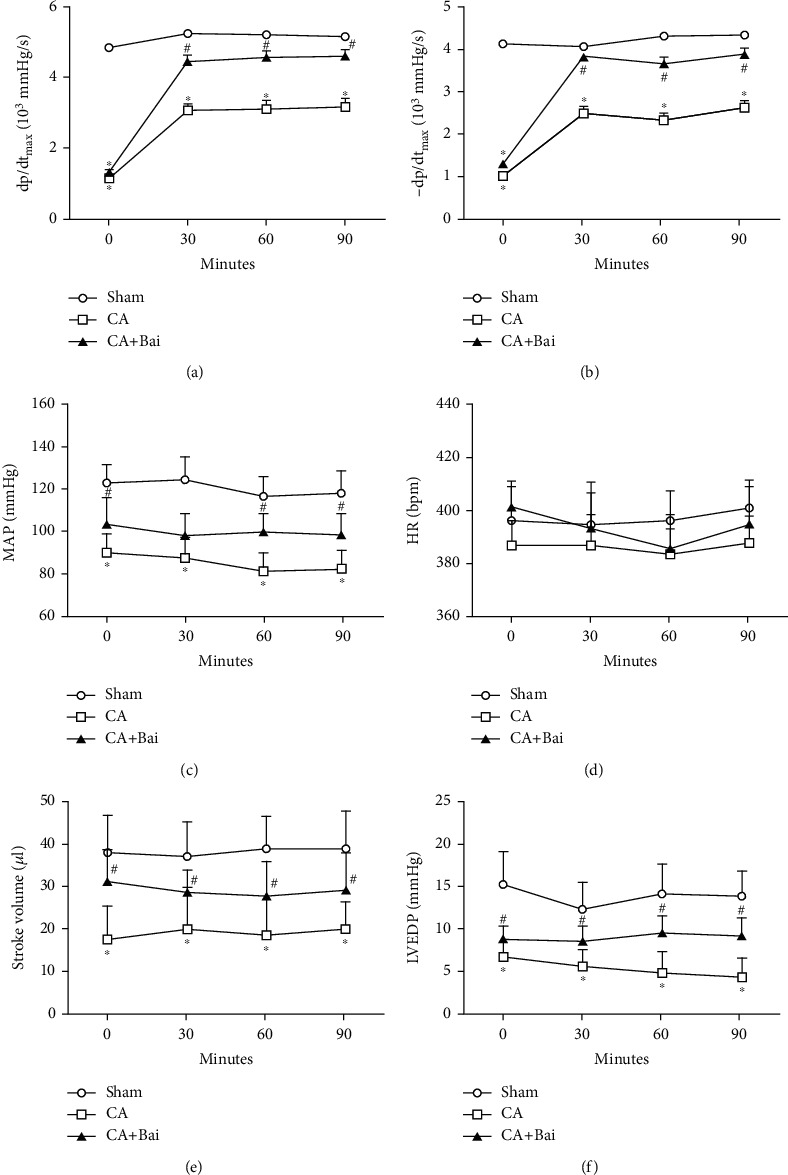
Baicalin (Bai) improved cardiovascular hemodynamics after cardiac arrest (CA). (a) Maximal rate of pressure development in the left ventricle (dp/dt_max_). (b) Maximal rate of pressure decay in the left ventricle (-dp/dt_max_). (c) Mean arterial blood pressure (MAP). (d) Heart rate (HR). (e) Stroke volume. (f) Left ventricular end-diastolic pressure (LVEDP). Data are presented as mean ± SEM. ^∗^*p* < 0.05 versus the sham group. ^#^*p* < 0.05 versus the CA group.

**Figure 3:**
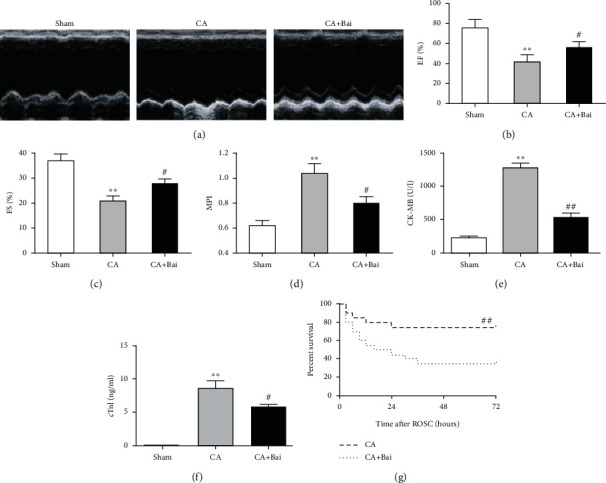
Baicalin (Bai) improved cardiac function and survival after cardiac arrest (CA). (a) Representative echocardiography images. (b) Left ventricular ejection fraction (EF). (c) Left ventricular fractional shortening (FS). (d) Myocardial performance index (MPI). (e) Serum creatine kinase MB (CK-MB) levels. (f) Serum cardiac troponin I (cTnI) levels. (g) Kaplan-Meier analyses of cumulative survival of rats during a 3-day follow-up after CA. Data are presented as mean ± SEM. ^∗∗^*p* < 0.01 versus the sham group. ^#^*p* < 0.05 and ^##^*p* < 0.01 versus the CA group.

To determine whether baicalin could be cardioprotective after CA, hemodynamics was examined after baicalin administration during the first 90 min of ROSC. Maximal positive and negative values of the instantaneous first derivative of left ventricular pressure (dp/dt_max_ and -dP/dt_max_), MAP, stroke volume, and left ventricular end-diastolic pressure (LVEDP) were all significantly depressed after ROSC. Baicalin did not affect the heart rate, while it indeed significantly improved CA-depressed postresuscitation cardiac hemodynamics (Figures 2(a)–2(f)). To further confirm the myocardial effects of baicalin after CA, we next performed transthoracic echocardiography, a noninvasive means of monitoring cardiac function. As shown in Figures 3(a)–3(c), a significant decrease in EF and FS was observed at 6 h after ROSC, which was significantly increased by baicalin treatment. Accordingly, MPI was significantly increased at 6 h after ROSC and baicalin treatment prevented this increase (Figures 3(a) and 3(d)). Serum CK-MB and cTnI are two primary markers of myocardial injury and are therefore used to further evaluate cardiac outcome. CK-MB and cTnI activities could be obviously detected in the rats subjected to CA. Moreover, baicalin treatment led to a significant decrease in the levels of CK-MB and cTnI activities (Figures 3(e) and 3(f)).

Next, we investigated whether baicalin affects animals' survival by analyzing survival curves. The 3-day survival experiment showed that 35% of the rats (7 of 20) in the CA group survived at day 3, which was much lower than that in the CA+Bai group (75%, 15 of 20) (Figure 3(f)). The Kaplan-Meier survival analysis showed that baicalin treatment significantly increased the 3-day survival time of the animals after ROSC.

### 3.2. Baicalin Treatment Attenuated Myocardial Injuries after ROSC

**Figure 4:**
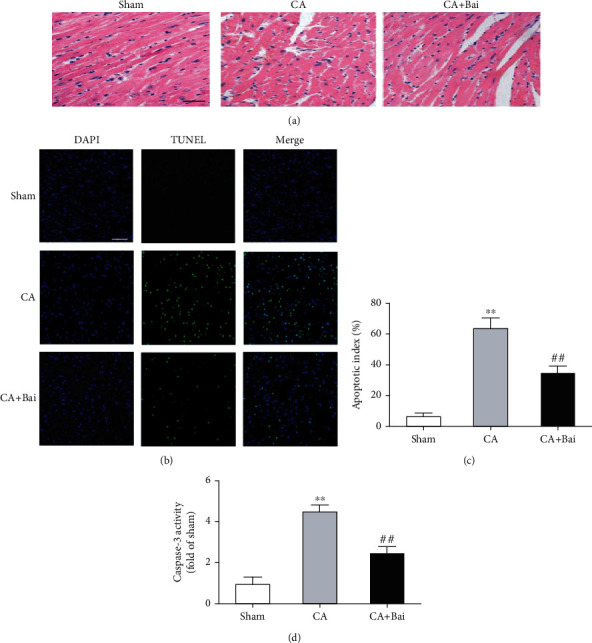
Baicalin (Bai) treatment reduced necrosis and apoptosis in the myocardium after cardiac arrest (CA). (a) Representative images of hematoxylin-eosin (HE) staining in 3 groups (×200). Scale bar = 20 *μ*m. (b) Representative images of terminal deoxynucleotide transferase-mediated dUTP-biotin nick-end labeling (TUNEL) staining in 3 groups. Scale bar = 50 *μ*m. (c) Apoptosis index. (d) Myocardial caspase-3 activity (fold of sham). Data are presented as mean ± SEM. ^∗∗^*p* < 0.01 versus the sham group. ^##^*p* < 0.01 versus the CA group.

To obtain more evidence on the beneficial effect of baicalin after CA, we assessed cardiac changes in pathological structure. HE staining revealed significant myocardial necrosis in the CA group, as observed by the disorders in the myocardial cells, the breakage in the myocardial fiber, and the irregular sizes of the cellular nucleus, which was significantly attenuated by baicalin treatment (Figure 4(a)). TUNEL staining showed apoptotic cells were significantly increased in the CA group compared with the sham group (9.7 ± 1.3 vs. 1.2 ± 0.3, *p* < 0.01) and this increase was significantly attenuated by baicalin treatment (Figures 4(b) and 4(c)). To further assess apoptosis in the myocardium, caspase-3 activity was measured at 72 h after CA treated with or without baicalin. A significant increase in caspase-3 activity was observed after CA, as expected, which was prevented by baicalin treatment (Figure 4(d)).

### 3.3. Baicalin Inhibited Drp1-Mediated Mitochondrial Fission and Improved Mitochondrial Function after CA

**Figure 5:**
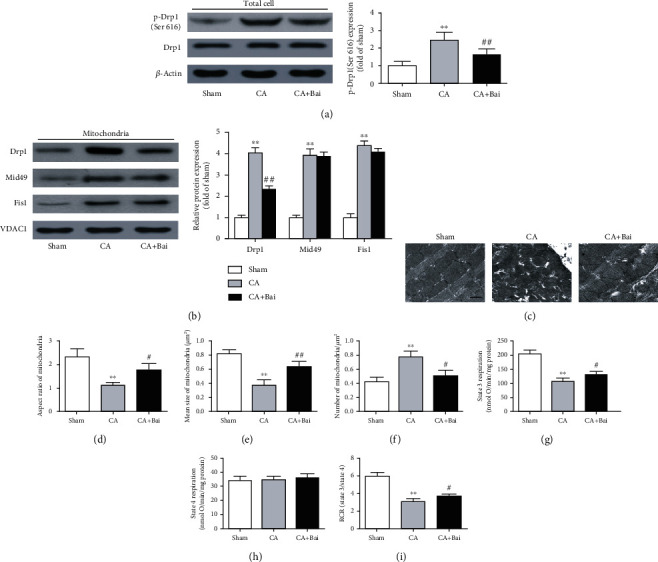
Baicalin (Bai) treatment prevented Drp1-mediated mitochondrial fission and improved mitochondrial function after cardiac arrest (CA). (a) Representative western blots and quantitative analysis of total Drp1, p-Drp1 (Ser 616), and *β*-actin protein expression. *β*-Actin served as a loading control. Quantification of p-Drp1 (Ser 616) expression was calculated as a ratio of p-Drp1 (Ser 616) levels to total Drp1 levels. (b) Representative western blots and quantitative analysis of mitochondrial Drp1, Mid49, Fis1, and VDAC1 protein expression. VDAC1 served as a mitochondrial loading control. (c) Representative transmission electron microscopic images from the myocardium (major finding is (d)–(f)). Scale bar = 1 *μ*m. *n* > 300 mitochondria/group. (d) The aspect ratio of mitochondria. (e) Mean size of mitochondria. (f) The number of mitochondria per *μ*m^2^. (g) State 3 respiration. (h) State 4 respiration. (i) Respiratory control ratio (RCR). Data are presented as mean ± SEM. ^∗∗^*p* < 0.01 versus the sham group. ^#^*p* < 0.05 and ^##^*p* < 0.01 versus the CA group.

We sought to study the underlying mechanism by which baicalin protects the myocardium and examined whether this protective effect was associated with mitochondrial fission. Mitochondrial fission is primarily controlled by Drp1; we thus first assessed changes in Drp1 expression. The results of western blotting showed that changes in the total expressions of Drp1 were not observed, while phosphorylation of Drp1 at serine 616 and translocation of Drp1 to the mitochondria were significantly increased after CA. Moreover, these increases were prevented by baicalin treatment (Figures 5(a) and 5(b)). To further investigate the possible mechanism, we tested the expression of Drp1 receptors that can recruit Drp1 to the mitochondrial outer membrane [29, 30]. A significant increase in the protein levels of Drp1 receptors (Mid49 and Fis1) was observed after CA, whereas this increase was not significantly attenuated by the treatment of baicalin (Figure 5(b)). Changes in mitochondrial morphology were detected by electron microscopy. Results in Figures 5(c)–5(f) showed that reduced aspect ratio and smaller mean size of mitochondria and increased number of mitochondria per *μ*m^2^ were clearly seen in the CA group compared with the sham group, which were significantly attenuated by baicalin treatment. Meanwhile, mitochondrial state 3 respiration and RCR in the heart were significantly decreased after CA and these were partly prevented with the administration of baicalin (Figures 5(g) and 5(i)).

### 3.4. Baicalin Prevented I/R-Induced Drp1-Mediated Mitochondrial Fission and H9c2 Cell Death

**Figure 6:**
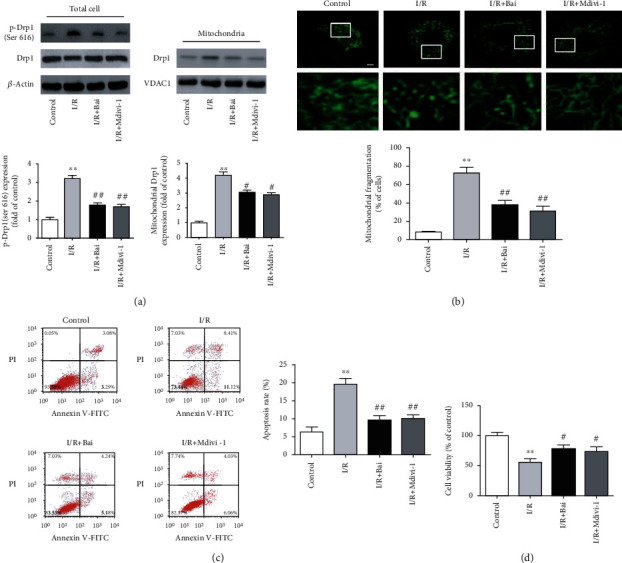
Both baicalin (Bai) and Mdivi-1 attenuated Drp1-mediated mitochondrial fission and cardiomyocyte injury induced by simulated ischemia/reperfusion (I/R). (a) Representative western blots of mitochondrial Drp1, p-Drp1 (Ser 616), VDAC1, and *β*-actin protein expression. VDAC1 and *β*-actin served as a mitochondrial and cytosolic loading control, respectively. Quantification of p-Drp1 (Ser 616) expression was calculated as a ratio of p-Drp1 (Ser 616) levels to total Drp1 levels. (b) Representative confocal photomicrographs showing mitochondrial morphology and the percentage of cells with fragmented mitochondria. Scale bar = 5 *μ*m. (c) Representative flow cytometric dot plots of cell apoptosis and quantitative analysis of apoptosis rate. (d) Cell viability (percentage of control). Data are presented as mean ± SEM. ^∗∗^*p* < 0.01 versus the sham group. ^#^*p* < 0.05 and ^##^*p* < 0.01 versus the CA group.

To further determine whether baicalin could inhibit Drp1-mediated mitochondrial fission in cardiac myocytes, H9c2 cells were pretreated with baicalin for 2 hours and cultured in experimental I/R. After testing the effects of baicalin at different concentrations in the I/R model, a final concentration of 20 *μ*M was used for the following experiments. As shown in Figure 6(a), the cell in I/R exhibited a significant increase of phosphorylation of Drp1 at serine 616 and Drp1 translocation to the mitochondria, whereas baicalin treatment prevented this increase. Next, we investigated whether baicalin inhibits I/R-induced mitochondrial fragmentation in H9c2 cells by visualizing mitochondrial morphology with MitoTracker Green probe. Elongated, thread-like mitochondria in interconnected networks were observed in the negative control group. After I/R, mitochondria became shorter and spherical in cells, indicating mitochondrial fragmentation occurred. Notably, treatment with baicalin attenuated I/R-induced mitochondrial fragmentation (Figure 6(b)). We further investigated whether baicalin could have a protective effect during I/R. As shown in Figure 6(c), cell apoptosis assessed by flow cytometry was significantly increased in I/R cells, and these increases were significantly attenuated by baicalin treatment. By contrast, the viability of H9c2 cells was significantly reduced after I/R and treatment with baicalin increased their viability (Figure 6(d)). Together, these results indicate that baicalin inhibited I/R-induced mitochondrial fission and cell death. Inhibition of Drp1 with 50 *μ*M Mdivi-1 for 1 h in vitro exerted similar protective effects against I/R-induced mitochondrial fission and cell death.

### 3.5. Baicalin Inhibited I/R-Induced ROS Production and Cytochrome c Release and Improved Mitochondrial Respiration in H9c2 Cells

**Figure 7:**
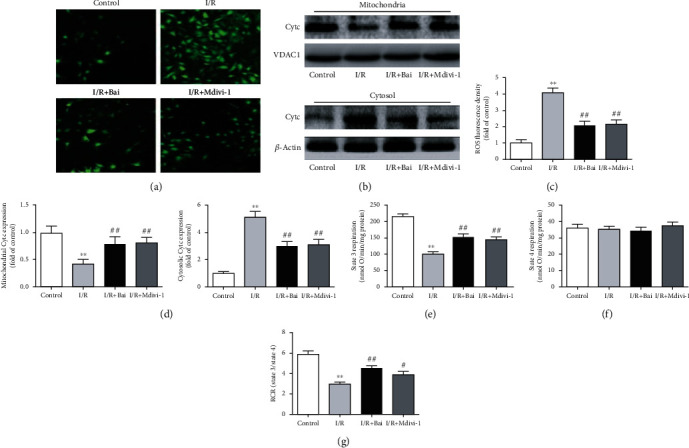
Both baicalin (Bai) and Mdivi-1 prevented reactive oxygen species (ROS) production, inhibited cytochrome c (Cytc) release, and improved mitochondrial respiration in H9c2 cells subjected to ischemia/reperfusion (I/R). (a) Representative fluorescent microscopy images of DCFH-DA staining. (b) Representative western blots of Cytc, VDAC1, and *β*-actin protein expression. VDAC1 and *β*-actin served as a mitochondrial and cytosolic loading control, respectively. (c) Fluorescence intensity quantification of DCFH-DA staining. (d) Quantitative analysis of mitochondrial and cytosolic Cytc protein expression. (e) State 3 respiration. (f) State 4 respiration. (g) Respiratory control ratio (RCR). Data are presented as mean ± SEM. ^∗∗^*p* < 0.01 versus the sham group. ^#^*p* < 0.05 and ^##^*p* < 0.01 versus the CA group.

Mitochondria are the primary source of cellular ROS production. Green DCF was detected using DCFH-DA probe to assess ROS production. The DCF fluorescence intensity was significantly higher in I/R cells than in the control cells (4-fold of control, *p* < 0.01), indicating an extensive production of ROS stimulated by I/R. Notably, baicalin and Mdivi-1 treatment significantly attenuated I/R-induced ROS production (Figures 7(a) and 7(c)). Excessive ROS can result in the release of proapoptotic factors especially cytochrome c in the mitochondria. We found that cytochrome c from mitochondria was largely released to cytosol in I/R cells compared with control cells, whereas baicalin and Mdivi-1 prevented cytochrome c release (Figures 7(b) and 7(d)). Next, we assessed the functional change of mitochondrial respiration in adult cardiomyocytes. As shown in Figures 7(e)–7(g), the state 3 respiration and RCR were significantly decreased in the I/R group compared with the control group (99.78 ± 8.5 vs. 213.12 ± 12.3 (*p* < 0.01) and 2.85 ± 0.35 vs. 5.9 ± 0.32 (*p* < 0.01)) and these decreases were significantly reversed by baicalin and Mdivi-1 treatment. However, no remarkable difference was reported in the state 4 respiration between each group.

### 3.6. Overexpression of Drp1 Abolished the Beneficial Effects of Baicalin in I/R-Induced Cell Injury

**Figure 8:**
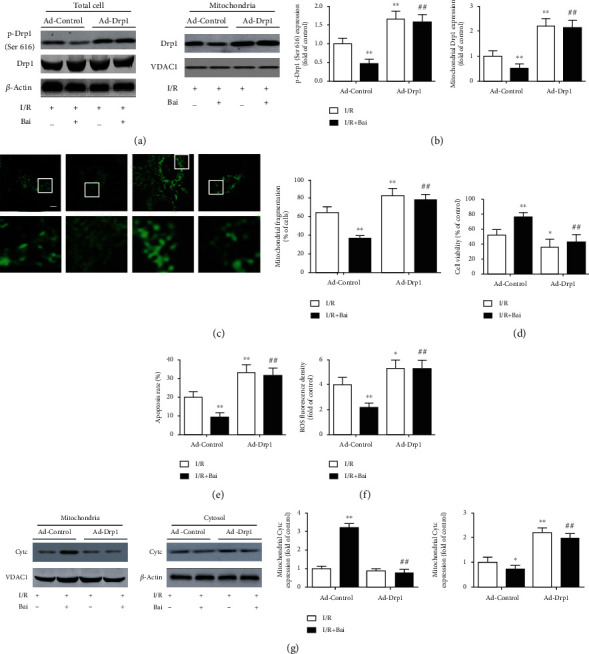
Adenoviral (Ad) overexpression of Drp1 blunted the effect of baicalin (Bai) on mitochondrial fission, cell injury, reactive oxygen species (ROS) production, and cytochrome c (Cytc) release induced by simulated ischemia/reperfusion (I/R). (a) Representative western blots of Drp1, p-Drp1 (Ser 616), *β*-actin, and VDAC1 protein expression. *β*-Actin served as a cytosolic or total protein loading control. VDAC1 served as a mitochondrial loading control. (b) Quantitative analysis of p-Drp1 (Ser 616) and mitochondrial Drp1 protein expression. Quantification of p-Drp1 (Ser 616) expression was calculated as a ratio of p-Drp1 (Ser 616) levels to total Drp1 levels. (c) Representative confocal photomicrographs showing mitochondrial morphology and quantitative analysis of the percentage of cells with fragmented mitochondria. Scale bar = 10 *μ*m. (d) Cell viability (percentage of control). (e) The apoptosis rate of H9c2 cells. (f) Fluorescence intensity quantification of ROS production. (g) Representative western blots and quantitative analysis of Cytc, VDAC1, and *β*-actin protein expression. VDAC1 and *β*-actin served as a mitochondrial and cytosolic loading control, respectively. Data are presented as mean ± SEM. ^∗^*p* < 0.05 and ^∗∗^*p* < 0.01 versus I/R with Ad-Control group. ^##^*p* < 0.01 versus I/R+Bai with Ad-Control group.

To further elucidate whether baicalin-afforded beneficial effects on I/R-induced cell injury are mediated by Drp1, we upregulated the Drp1 expression by infecting H9c2 cells with adenoviruses expressing Drp1. The role of Drp1 in the I/R model was confirmed in promoting mitochondrial fragmentation, cardiomyocyte death, ROS production, and cytochrome c release (Figures 8(a)–8(g)). The protective effects of baicalin on I/R-induced cell injury were largely abolished by Drp1 overexpression. These results demonstrated that baicalin might protect cardiomyocytes against I/R-induced cell damage mainly through downregulating Drp1.

## 4. Discussion

In the current study, the cardioprotective role of baicalin associated with mitochondrial function and the underlying mechanism was explored. Our findings indicated that baicalin could improve survival and cardiac outcome including increased LV function and reduced myocardial enzyme activities. Baicalin also ameliorated CA-induced myocardial injury by reducing cell necrosis and apoptosis in vivo. The possible mechanism is that baicalin could alleviate mitochondrial dysfunction through suppressing Drp1-mediated mitochondrial fission.

CA is a sudden and unexpected event and usually caused by cardiogenic or other acute diseases, which consist of serious electrolyte disorder, acid-base imbalance, trauma, and drowning [31]. The initial CA is difficult to prevent due to the absence of reliable early identification; thus, great emphasis is placed on the effective intervention after CA occurs. Herein, our results show that daily administration of baicalin for 28 days before CA attenuates myocardial injury induced by CA, supporting that long-term daily use of baicalin can serve as a promising therapeutic strategy to lower myocardial injury in CA. This intervention could be practicable in future clinical application because baicalin has been demonstrated to provide good validity in treating obesity [32], hyperglycemia [33], and hyperglycemia-induced vascular complications such as endothelial dysfunction [34], which are all vital factors of increased risk for sudden CA. In addition, short-term intervention of baicalin for 7 days has been shown to be cardioprotective in myocardial infarction [35]; however, further study is required to determine whether short-term administration of baicalin is effective in reducing myocardial injury after CA.

Post-CA myocardial injury is a major medical challenge, and the effective strategies to treat it are still limited [36]. Mitochondria are essential organelles that are especially abundant in the myocardium to supply the cell with energy. The myocardium mainly depends on mitochondria for its function [37]. This dependence has made it possible to focus on mitochondrial dysfunction as a therapeutic target after CA. Targeting the mitochondrial dysfunctions has been demonstrated to be a promising intervention. Our recent study demonstrated that exogenous carbon monoxide treatment could improve neurological outcome and attenuate neuronal death by promoting mitochondrial autophagy, which maintained mitochondrial function in a similar animal model of CA [23]. Pharmacological inhibition of mitochondrial permeability transition pore opening was also shown to attenuate the post-CA syndrome and improve short-term cardiac outcomes [6]. In the present study, we found that the administration of baicalin attenuated myocardial injury via preventing mitochondrial dysfunction.

Accumulating evidence has indicated that imbalance of mitochondrial fusion and fission has been implicated in the regulation of the myocardial I/R injury including CA-induced injury [9, 12, 38]. In this study, enhanced mitochondrial fission was observed in the myocardial cell I/R injury, which is accompanied by the increase of ROS production, the release of cytochrome c from mitochondria, and the decline of mitochondrial respiratory function. Inhibition of mitochondrial fission by Drp1 inhibition with Mdivi-1 decreased ROS production, inhibited mitochondrial cytochrome c release, and improved mitochondrial respiratory function. Based on these results, we concluded that mitochondrial fission lies upstream of mitochondrial damage. This was consistent with previous discoveries that identified mitochondrial fission as the pathogenesis for myocardial acute I/R injury. Evidence showed that mitochondria are not only the major site of ROS production but also the major targets of ROS under stress [39]. Excessive ROS production can trigger mitochondrial dysfunction including the decrease in ATP generation [40], damage of respiratory chain activity [41], promoting mitochondrial membrane permeabilization, and immediately followed by the release of cytochrome c [42]. Drp1-mediated mitochondrial fission has been demonstrated to involve mitochondrial ROS production during induced cell death [43, 44]. Thus, ROS may be an important intermediate link connecting mitochondrial fission abnormality and dysfunction in the pathological process of myocardial I/R injury.

Drp1 is recruited from the cytosol onto the mitochondrial outer membrane to initiate mitochondrial fission [45]. The present work indicates that Drp1 plays an important role in the protective effect of baicalin. Researches have shown that Drp1 activities are mainly regulated by posttranslational modification including phosphorylation, proteolysis, or sumoylation [46]. During myocardial I/R injury, phosphorylation of Drp1 at serine 616 has been demonstrated to increase Drp1 enzyme activities and contribute to cell death [47]. Therefore, the beneficial effects elicited by baicalin treatment may be through inhibiting phosphorylation at its serine 616 in the present study, thereby reducing myocardial injury and eventually improving cardiac function and survival. Previous studies indicated that related Drp1 receptor proteins such as Fis1 and MiD49 could recruit Drp1 to the mitochondrial outer membrane [29, 30]. Fis1 and MiD49 might be involved in myocardial injury after CA; however, our results showed that baicalin failed to affect their protein expression, implying these receptor proteins do not appear to be targets of baicalin.

To our knowledge, this is the first report that investigated the effect of baicalin on the model of CA in vivo and in vitro. Baicalin has been widely utilized in the pharmaceutical and food industries due to its excellent bioactivities [48]. Recent studies have been attracted to study the effects of baicalin in cardiovascular diseases. Baicalin was shown to alleviate hypertension-associated intestinal barrier impairment by enhancing microbial production of short-chain fatty acids [49]. Baicalin also reduced cardiomyocyte apoptosis under hypoxic condition via activating the Nrf2/HO-1 pathway [50]. Recently, the combined application of baicalin and berberine was found to relax blood vessels through the K_ATP_ channel and the voltage-dependent Ca2+ channel [51]. Moreover, a randomized, double-blind, placebo-controlled clinical trial has revealed the clinical efficacy in patients with coronary artery disease and rheumatoid arthritis is mediated via anti-inflammation and antihyperlipidemia [52]. Herein, our results showed a novel finding that long-term baicalin treatment affords potent cardioprotection after CA through suppressing Drp1 activation and subsequent mitochondrial fission process.

In the present study, we found that through inhibiting the phosphorylation at serine 616 and translocation of Drp1 and excessive fission of mitochondria, baicalin potently prevented dysfunction of mitochondria and conferred cardioprotective effect after CA. In summary, our study provided the first evidence that inhibiting Drp1-mediated mitochondrial fission might be one of the possible mechanisms of baicalin in preventing CA-induced myocardial injury, which shed insights into the role of baicalin as a new prospective agent against CA.

## Data Availability

All data supporting the conclusions of this article are included in this article.

## Supplementary Materials

Supplementary Materials Supplementary Figure 1: effects of different doses of baicalin (Bai) on cardiovascular hemodynamics after cardiac arrest (CA). (A) Maximal rate of pressure development in the left ventricle (dp/dt_max_). (B) Maximal rate of pressure decay in the left ventricle (-dp/dt_max_). (C) Mean arterial blood pressure (MAP). (D) Heart rate (HR). (E) Stroke volume. (F) Left ventricular end-diastolic pressure (LVEDP). Data are presented as mean ± SEM. ^#^*p* < 0.05 versus the CA group.
