# Supplementary material for: Baicalin Improves Cardiac Outcome and Survival by Suppressing Drp1-Mediated Mitochondrial Fission after Cardiac Arrest-Induced Myocardial Damage
Source: Oxid Med Cell Longev. 2021 Feb 1;2021:8865762. doi: 10.1155/2021/8865762 (PMC7870315; doi:10.1155/2021/8865762)
Supplement: Supplementary Materials — Supplementary Figure 1: effects of different doses of baicalin (Bai) on cardiovascular hemodynamics after cardiac arrest (CA). (A) Maximal rate of pressure development in the left ventricle (dp/dtmax). (B) Maximal rate of pressure decay in the left ventricle (-dp/dtmax). (C) Mean arterial blood pressure (MAP). (D) Heart rate (HR). (E) Stroke volume. (F) Left ventricular end-diastolic pressure (LVEDP). Data are presented as mean ± SEM. #p < 0.05 versus the CA group. [file 8865762.f1.zip › ID 8865762 figures.pptx]

## Slide 1
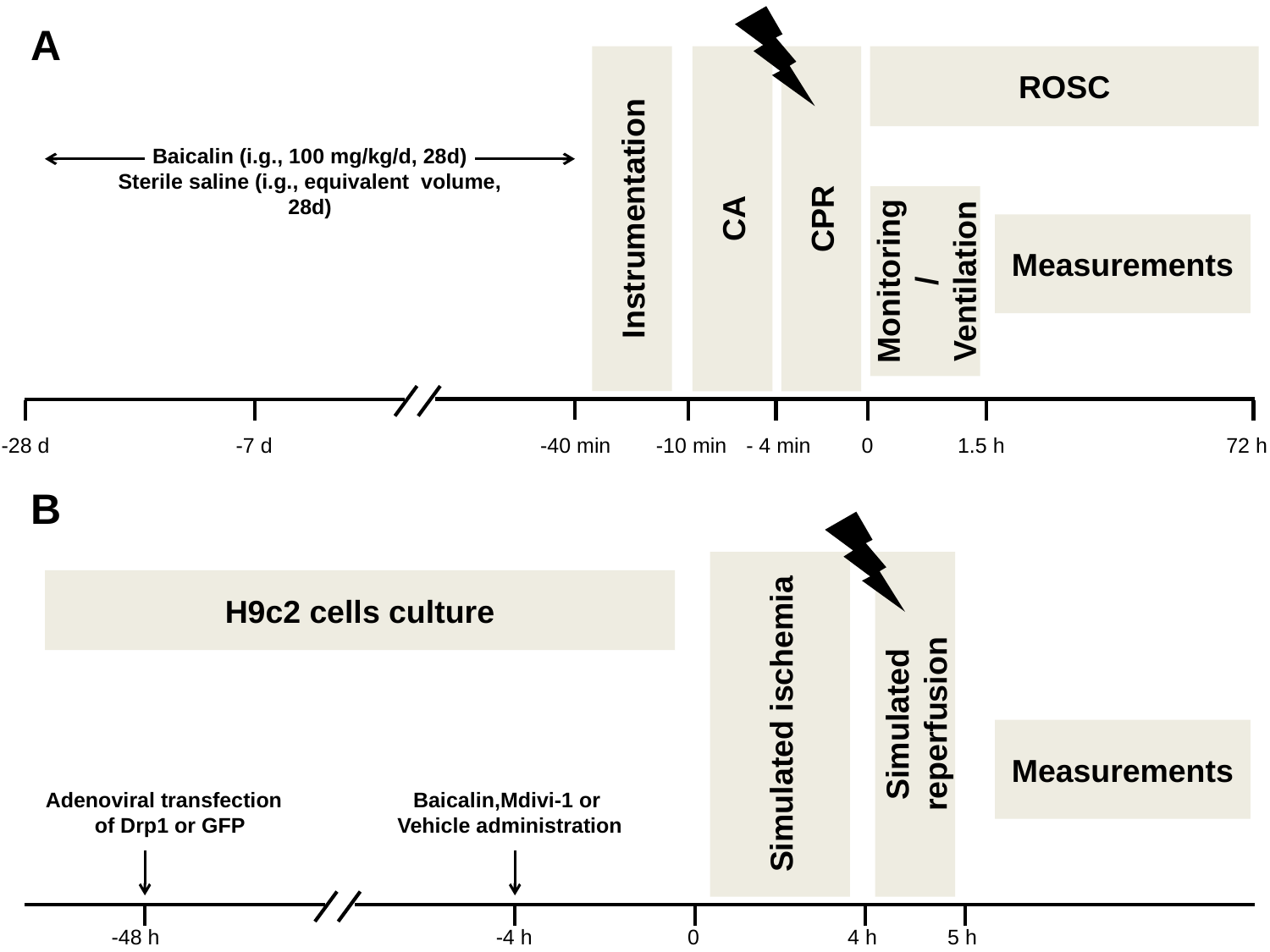

ROSC
Instrumentation
CA
CPR
Baicalin (i.g., 100 mg/kg/d, 28d)
Sterile saline (i.g., equivalent volume, 28d)
Monitoring/
Ventilation
Measurements
-28 d
-7 d
-40 min
-10 min
- 4 min
0
1.5 h
72 h
A
H9c2 cells culture
B
Simulated ischemia
Simulated reperfusion
Measurements
Adenoviral transfection
of Drp1 or GFP
Baicalin,Mdivi-1 or
Vehicle administration
-48 h
-4 h
0
4 h
5 h

## Slide 2
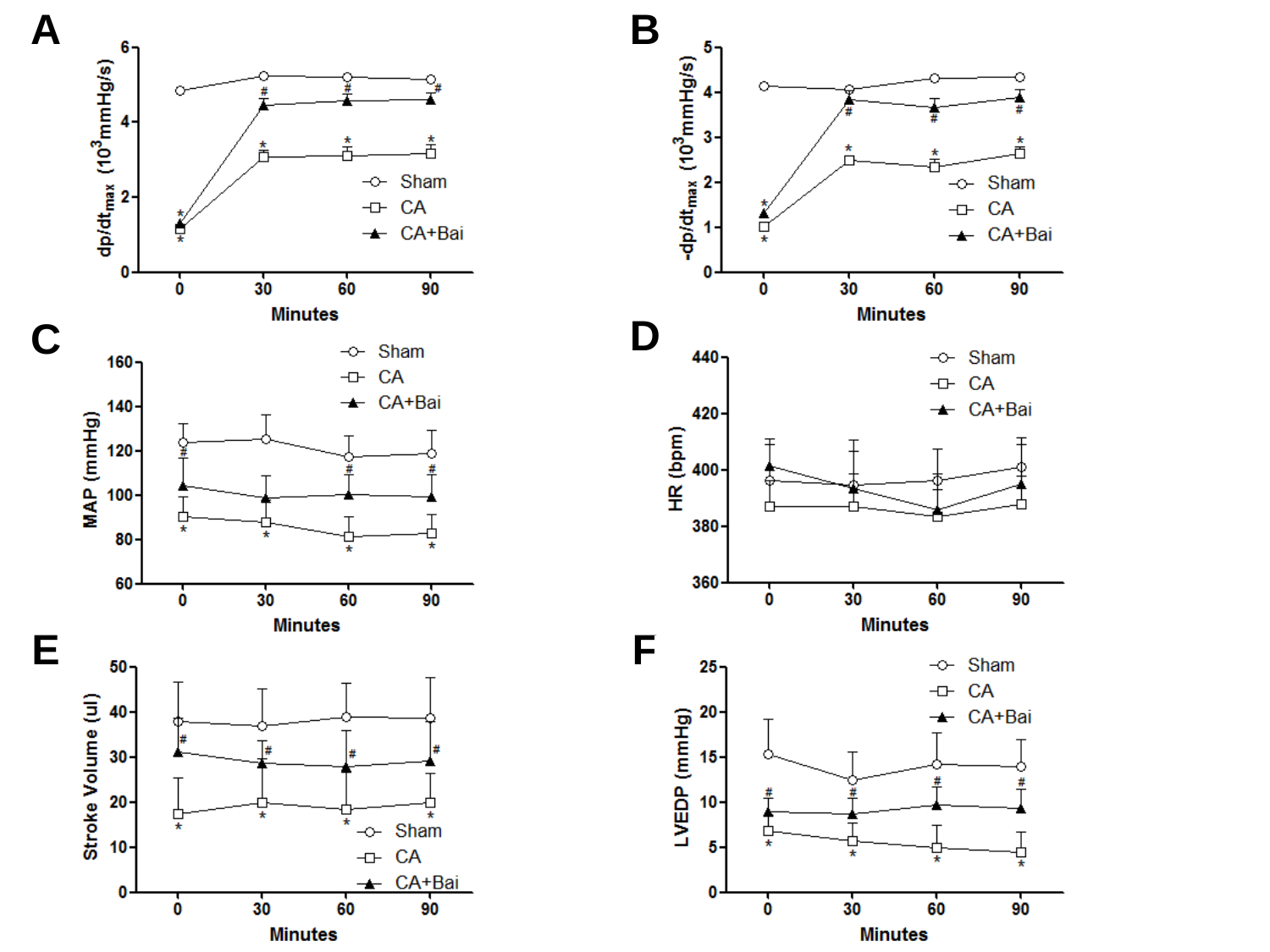

A
B
D
C
E
F

## Slide 3
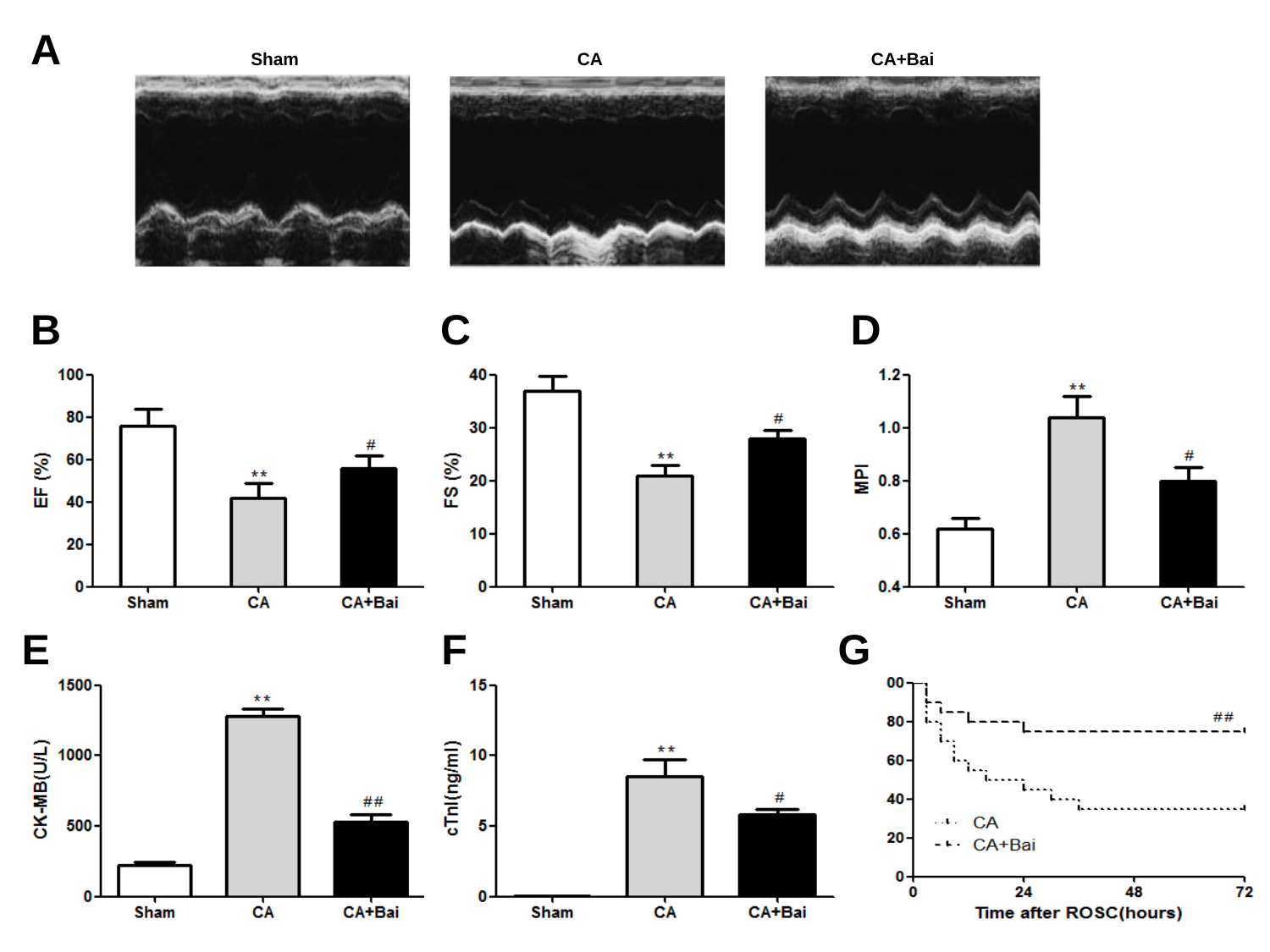

A
Sham
CA
CA+Bai
B
C
D
E
F
G

## Slide 4
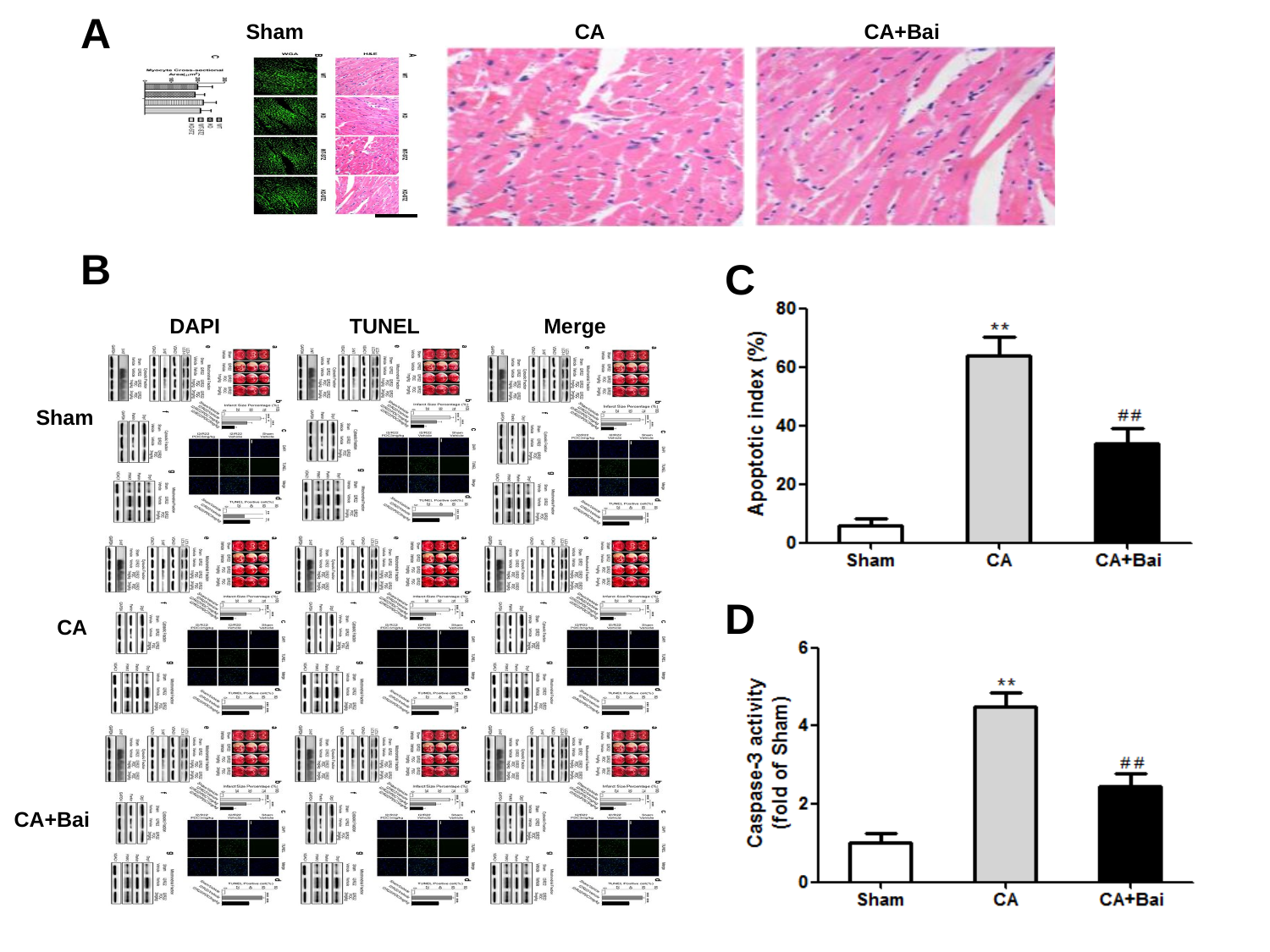

A
Sham
CA
CA+Bai
B
C
DAPI
TUNEL
Merge
Sham
D
CA
CA+Bai

## Slide 5
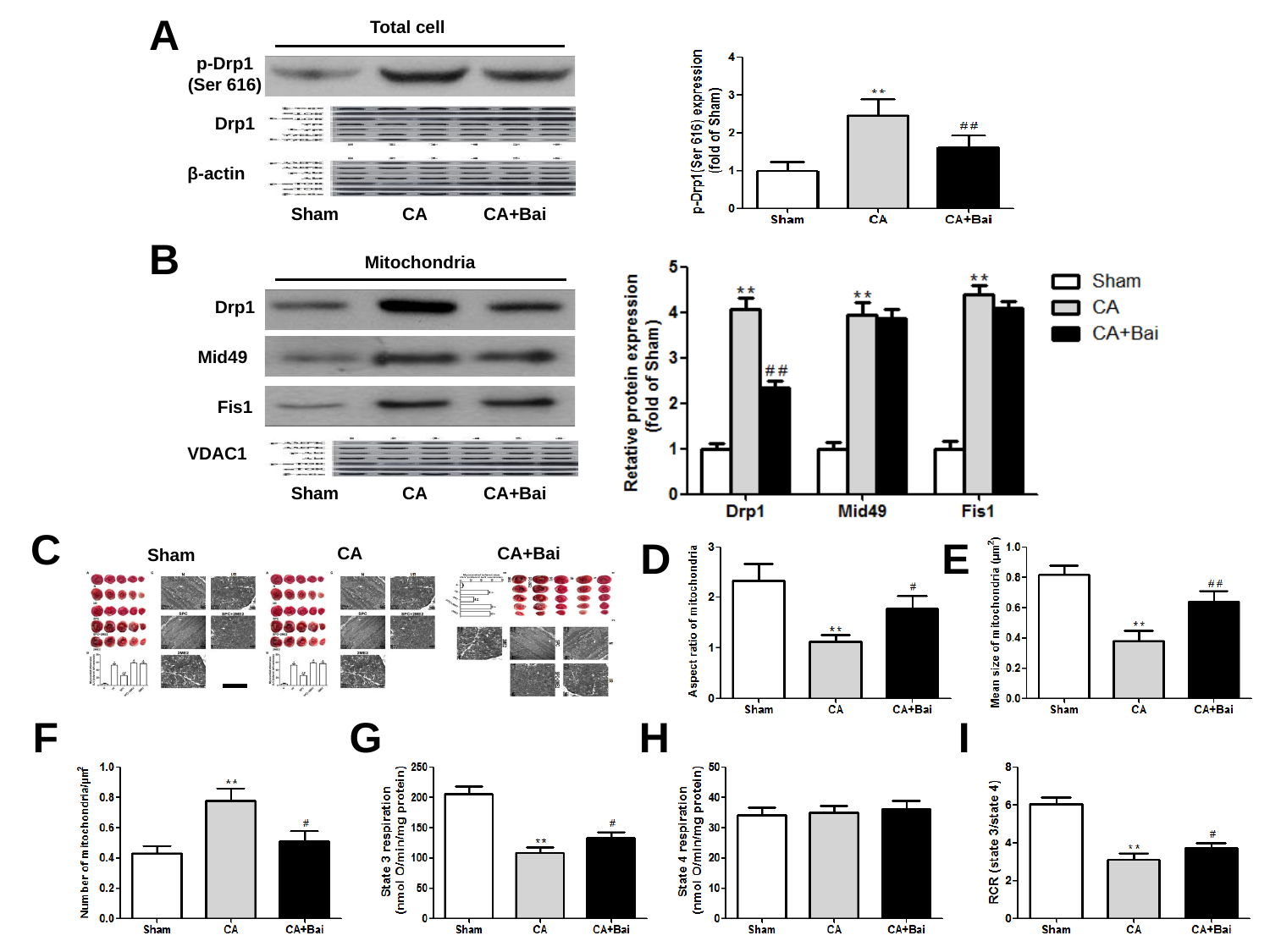

A
Total cell
p-Drp1
(Ser 616)
Drp1
β-actin
Sham
CA
CA+Bai
B
Mitochondria
Drp1
Mid49
Fis1
VDAC1
Sham
CA
CA+Bai
C
D
E
CA
CA+Bai
Sham
F
G
H
I

## Slide 6
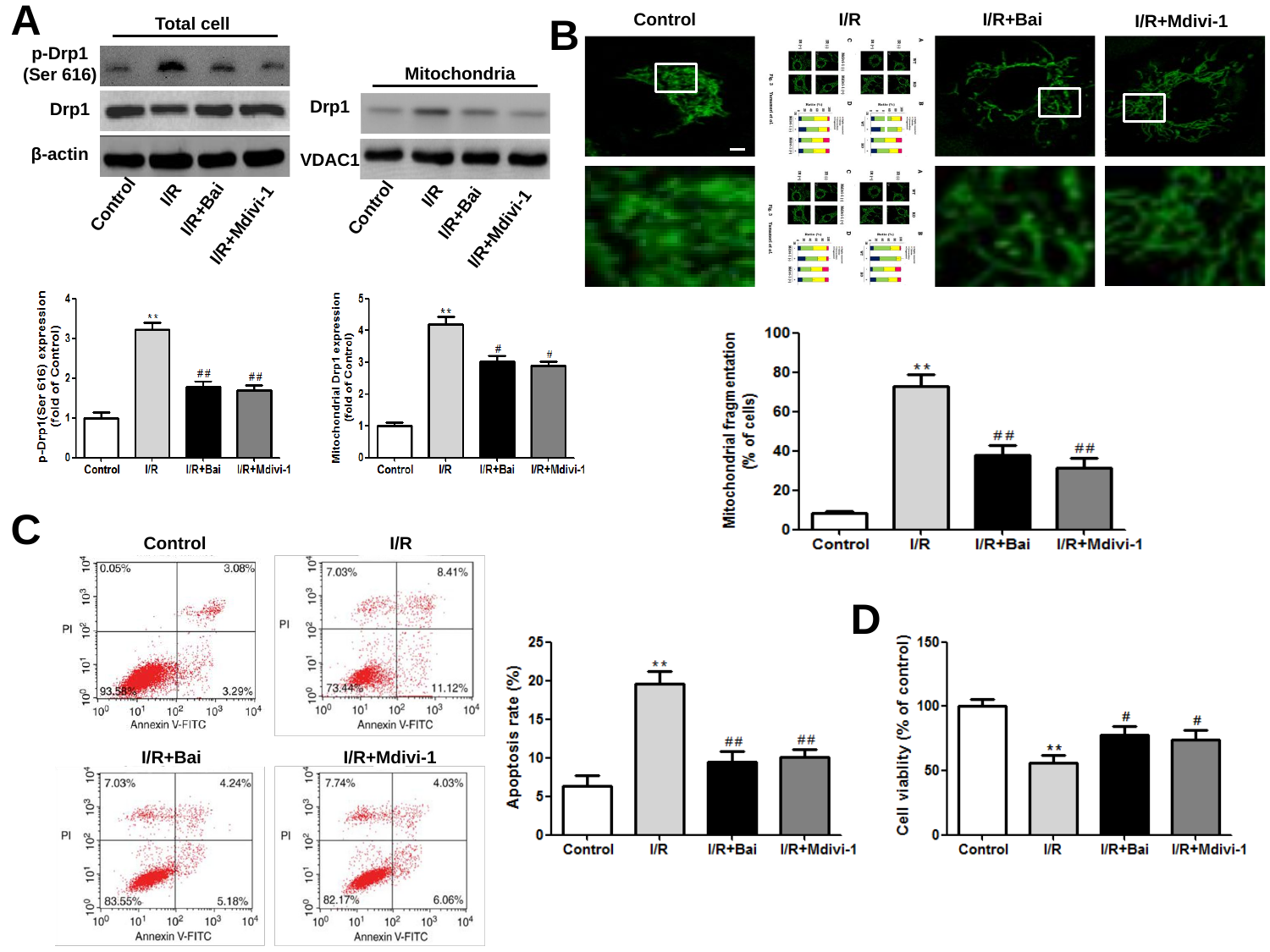

A
I/R+Mdivi-1
Control
I/R
I/R+Bai
B
Total cell
p-Drp1
(Ser 616)
Mitochondria
Drp1
Drp1
β-actin
VDAC1
I/R
I/R
Control
Control
I/R+Bai
I/R+Bai
I/R+Mdivi-1
I/R+Mdivi-1
C
Control
I/R
D
I/R+Bai
I/R+Mdivi-1

## Slide 7
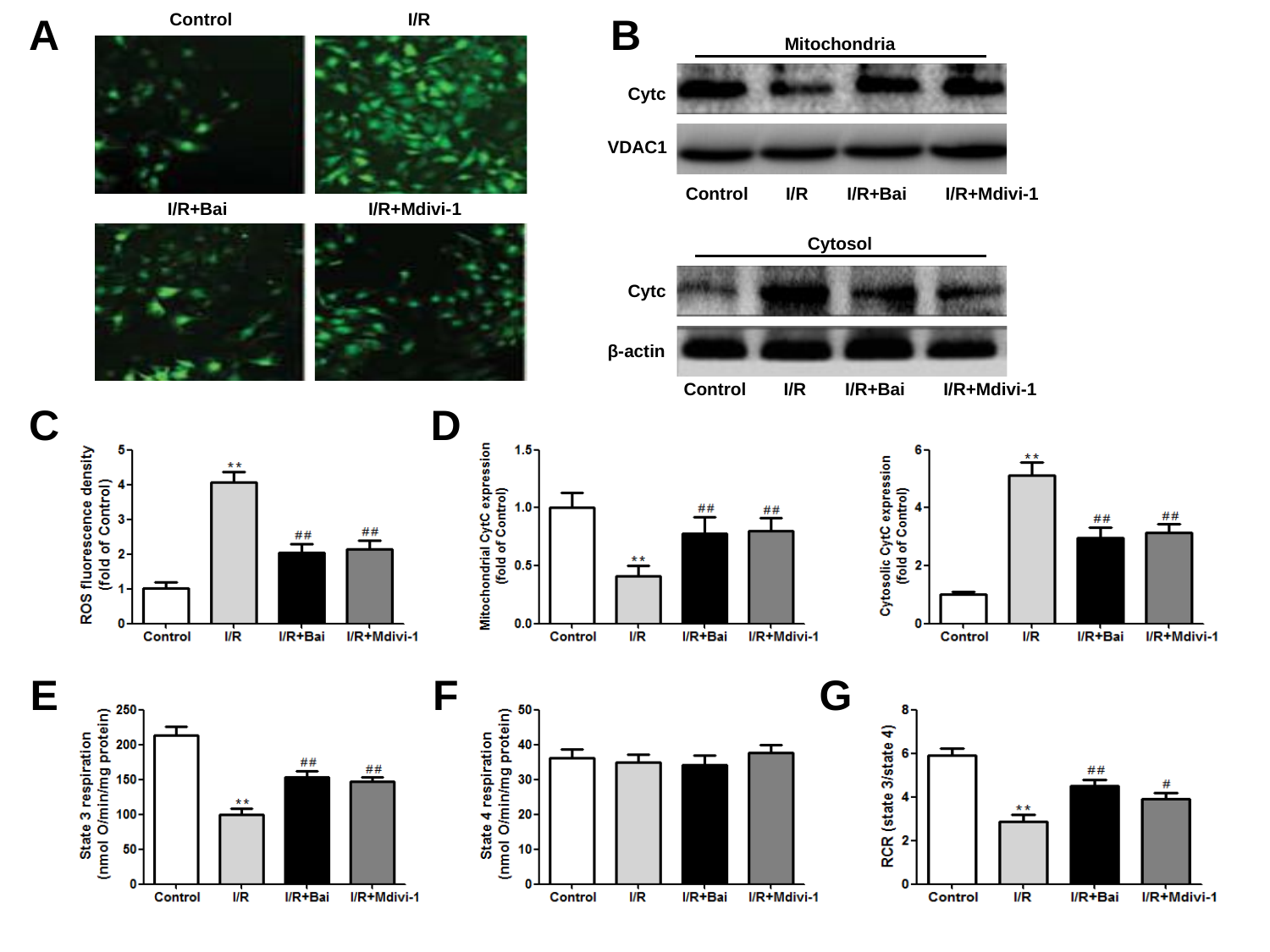

Control
I/R
A
B
Mitochondria
Cytc
VDAC1
Control
I/R
I/R+Bai
I/R+Mdivi-1
I/R+Bai
I/R+Mdivi-1
Cytosol
Cytc
β-actin
Control
I/R
I/R+Bai
I/R+Mdivi-1
C
D
E
F
G

## Slide 8
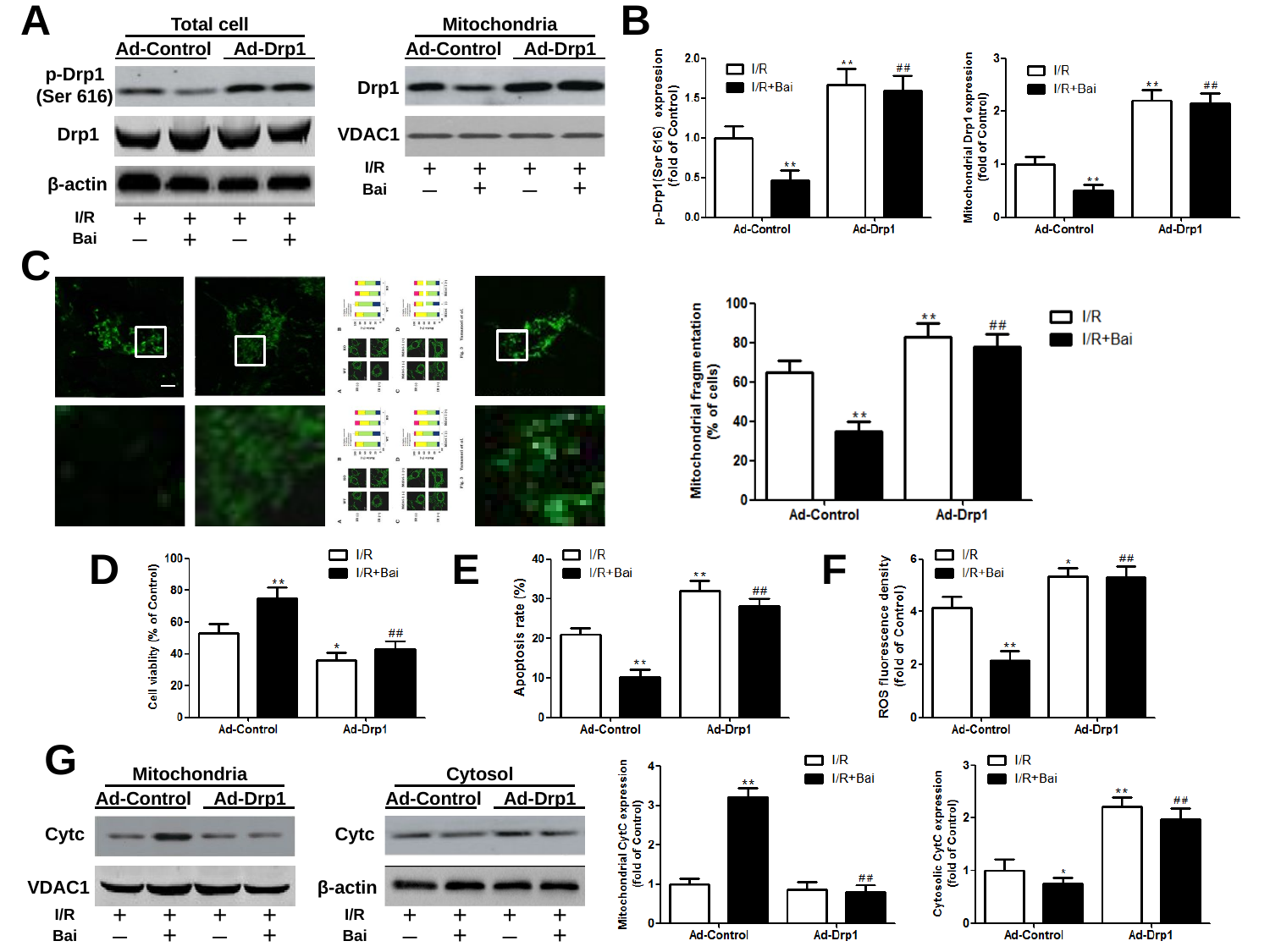

A
B
Total cell
Mitochondria
Ad-Control
Ad-Drp1
Ad-Control
Ad-Drp1
p-Drp1
(Ser 616)
Drp1
Drp1
VDAC1
+
+
+
+
I/R
_
_
β-actin
+
+
Bai
+
+
+
+
I/R
_
_
+
+
Bai
C
D
E
F
G
Mitochondria
Cytosol
Ad-Control
Ad-Drp1
Ad-Control
Ad-Drp1
Cytc
Cytc
VDAC1
β-actin
+
+
+
+
+
+
+
+
I/R
I/R
_
_
_
_
+
+
+
+
Bai
Bai
