# Supplementary figures and images for: Baicalin Improves Cardiac Outcome and Survival by Suppressing Drp1-Mediated Mitochondrial Fission after Cardiac Arrest-Induced Myocardial Damage
Source: Oxid Med Cell Longev. 2021 Feb 1;2021:8865762. doi: 10.1155/2021/8865762 (PMC7870315; doi:10.1155/2021/8865762)

## Slide 1
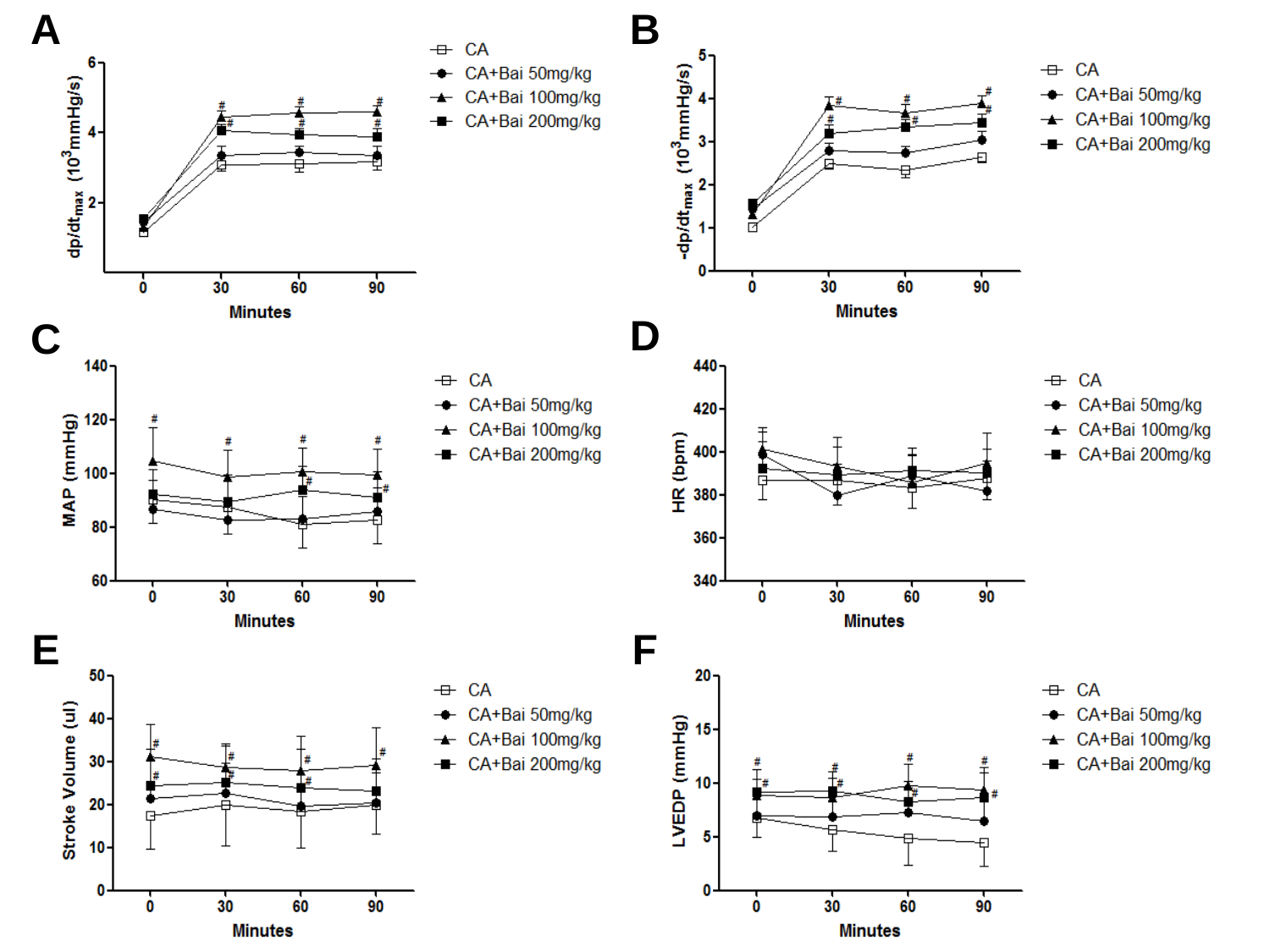

A
B
D
C
E
F

Supplement: Supplementary Materials — Supplementary Figure 1: effects of different doses of baicalin (Bai) on cardiovascular hemodynamics after cardiac arrest (CA). (A) Maximal rate of pressure development in the left ventricle (dp/dtmax). (B) Maximal rate of pressure decay in the left ventricle (-dp/dtmax). (C) Mean arterial blood pressure (MAP). (D) Heart rate (HR). (E) Stroke volume. (F) Left ventricular end-diastolic pressure (LVEDP). Data are presented as mean ± SEM. #p < 0.05 versus the CA group. [file 8865762.f1.zip › supplementary figure 1.pptx]
